# Phase-specific supplementation of a ternary emulsifier blend improves growth performance and nutrient utilization in broilers fed reduced-energy diets

**DOI:** 10.14202/vetworld.2026.3231-3245

**Published:** 2026-07-21

**Authors:** Phongthorn Kongmun, Sombat Prasongsook, Dirk Westphal, Sandor Zsarnoczay, Theerawit Poeikhampha

**Affiliations:** 1 Department of Animal Science, Faculty of Agriculture, Kasetsart University, Ngam Wong Wan, Bangkok 10900, Thailand.; 2 Berg-Schmidt Asia Pte Ltd, 1 North Buona Vista Link #10-06 Elementum, 139691, Singapore.

**Keywords:** broiler chickens, carcass yield, emulsifier supplementation, growth performance, lipid digestion, nutrient utilization, phase-specific feeding, reduced-energy diet

## Abstract

**Background and Aim:**

Dietary energy is the most expensive nutrient of broiler feed, and reducing metabolizable energy (ME) while maintaining performance is a major objective in poultry nutrition. Emulsifiers enhance lipid digestion and may compensate for reduced dietary energy. This study evaluated the effects of a novel ternary emulsifier blend consisting of sorbitan esters, lysolecithin, and de-oiled lecithin applied through a phase-specific supplementation strategy on growth performance, nutrient utilization, and carcass characteristics of broilers fed reduced-energy diets.

**Materials and Methods:**

A total of 750 one-day-old Ross 308 male broiler chicks were randomly assigned to five dietary treatments with six replicates of 25 birds each. Treatments included a positive control with standard ME, a negative control with ME reduced by 150 kcal/kg, and three emulsifier-supplemented reduced-energy diets: 250 g/ton emulsifier, 200 g/ton emulsifier, and a phase-specific strategy consisting of 250 g/ton during the starter and grower phases followed by 200 g/ton during the finisher phase. Birds were reared for 35 days. Growth performance, feed efficiency, feed cost per gain, gastrointestinal retention time, nutrient digestibility, apparent ME, fecal characteristics, and carcass traits were evaluated.

**Results:**

During the starter phase, emulsifier supplementation significantly improved final body weight and feed conversion ratio compared with the reduced-energy control (p < 0.05). Birds receiving the phase-specific strategy achieved body weight and feed efficiency comparable to those fed the standard-energy diet. During the grower phase, supplementation with 200 or 250 g/ton comparedof emulsifier increased body weight relative with the reduced-energy control (p = 0.04). However, no significant differences were observed among treatments for overall performance from 1 to 35 days. Emulsifier supplementation significantly reduced gastrointestinal retention time compared with the standard-energy control (p = 0.01). Apparent ME values were numerically higher in emulsifier-supplemented groups, although differences were not statistically significant. Carcass yield tended to improve with emulsifier supplementation, whereas carcass component proportions and abdominal fat deposition remained unaffected.

**Conclusion:**

The ternary emulsifier blend partially compensated for reduced dietary energy, particularly during the starter and grower phases. A phase-specific supplementation approach using higher inclusion levels during early growth and lower levels during finishing appears to be an effective and practical strategy for optimizing emulsifier use in reduced-energy broiler diets while maintaining productive performance.

## INTRODUCTION

Dietary energy is a fundamental and economically important component of broiler feed formulation because of its substantial influence on production costs, growth performance, feed efficiency, and carcass quality. Energy is typically the most expensive nutrient in poultry diets and often accounts for the largest proportion of feed-related expenditures [1]. Optimizing dietary energy levels can improve profitability by enhancing feed conversion ratio (FCR) and growth rate while reducing feed intake (FI) and associated production costs. Furthermore, precise energy formulation may reduce environmental impacts through lower nitrogen excretion, thereby supporting sustainable poultry production systems [2]. Reducing dietary energy while supplementing diets with emulsifiers has emerged as an effective nutritional strategy to maintain or improve growth performance, nutrient digestibility, and feed efficiency while lowering feed costs. Emulsifiers, including lecithin-based and synthetic compounds, improve fat emulsification and absorption, thereby compensating for the lower energy density of reduced-energy diets [3]. Feed emulsifiers function by reducing the size of dietary fat globules, increasing their surface area for enzymatic hydrolysis by intestinal lipase, and promoting micelle formation, which facilitates lipid transport across the intestinal epithelium. Consequently, emulsifiers improve fat utilization and enhance the absorption of fat-soluble vitamins [4].

Numerous studies have demonstrated that emulsifier supplementation can improve growth performance, nutrient digestibility, and feed efficiency in broilers and laying hens fed energy-restricted diets [5, 6]. Enhanced ileal digestibility of crude fat, dry matter, and energy, together with increased apparent metabolizable energy (AME), further demonstrates the beneficial effects of emulsifiers on nutrient utilization [4]. In addition, emulsifiers may modulate lipid metabolism by reducing serum cholesterol and triglyceride concentrations and regulating the expression of genes associated with lipid utilization [7, 8]. Although improvements in carcass traits have been inconsistent, with only minor effects reported on dressing percentage, liver weight, and breast muscle characteristics, the principal benefits of emulsifier supplementation are associated with enhanced growth and metabolic efficiency rather than changes in carcass composition [5, 6]. These findings highlight the potential of emulsifiers to support sustainable poultry production by improving nutrient utilization and reducing feed costs. However, their biological efficacy and economic value depend on several factors, including emulsifier type, composition, dietary energy level, and feeding strategy.

The efficacy of feed emulsifiers in broiler nutrition is influenced by their chemical characteristics and mode of application. Lecithin, a naturally derived emulsifier obtained primarily from plant sources, has been widely recognized for its role in enhancing lipid metabolism, reducing serum cholesterol concentrations, and improving intestinal morphology, all of which contribute to improved nutrient absorption and physiological health in broilers [9]. Furthermore, lecithin supplementation has been associated with a reduced incidence of fatty liver hemorrhagic syndrome in laying hens, indicating broader physiological benefits. In contrast, synthetic emulsifiers have shown particular effectiveness in reduced-energy diets, where they improve fat digestibility, body weight gain, and FCR [10]. Moreover, combinations of synthetic emulsifiers with monoglycerides and lysolecithin have been reported to improve litter quality and welfare indicators, including footpad condition [11–13]. Comparative studies suggest that although synthetic emulsifiers may provide greater improvements in growth efficiency, lecithin-based emulsifiers may offer additional physiological benefits, including improved gut morphology and plasma lipid profiles [14]. Therefore, the selection of an appropriate emulsifier should align with specific production objectives, as lecithin-based products primarily support physiological health, whereas synthetic emulsifiers may be more effective at enhancing productive performance under suboptimal dietary energy conditions.

Dietary emulsifier supplementation has also been reported to influence carcass yield and fat deposition in broilers, although the magnitude of these responses depends on the type of emulsifier, dietary fat source, and dietary energy concentration. By enhancing lipid digestion and absorption, emulsifiers may improve nutrient availability for tissue accretion, thereby influencing carcass characteristics. The present study evaluated a ternary emulsifier blend composed of sorbitan esters, lysolecithin, and de-oiled lecithin. Sorbitan esters are non-ionic surfactants derived from sorbitol and fatty acids that facilitate dietary fat emulsification, thereby improving lipase accessibility and micelle formation within the gastrointestinal tract [15]. Lysolecithin, a hydrolyzed derivative of lecithin rich in lysophospholipids such as lysophosphatidylcholine, possesses superior emulsifying properties and has been associated with improved intestinal morphology, increased villus height, enhanced nutrient transport, and improved absorption of fat-soluble vitamins [16]. In addition, lysolecithin contributes to gut health and membrane permeability. De-oiled lecithin, a concentrated phospholipid fraction with reduced oil content, provides moderate emulsification capacity while improving lipid dispersion, nutrient digestibility, and feed efficiency. It also offers technological advantages, including improved heat stability and ease of feed manufacturing [17]. Collectively, these emulsifying agents may enhance lipid utilization, improve growth performance, and support carcass development in broilers fed reduced-energy diets.

Previous studies have primarily focused on single-component emulsifiers or binary emulsifier combinations, particularly lecithin, lysolecithin, and synthetic emulsifiers, administered at fixed inclusion rates throughout the production cycle [10–14]. Although these studies have demonstrated improvements in fat digestibility and growth performance, limited information is available on the efficacy of ternary emulsifier systems combining sorbitan esters, lysolecithin, and de-oiled lecithin. Furthermore, little attention has been paid to phase-specific supplementation strategies that adjust emulsifier inclusion levels in line with the physiological development of broilers. Young broilers exhibit limited endogenous bile secretion and lípid-digestion capacity, whereas digestive efficiency progressively improves with age. Therefore, a constant level of emulsifier inclusion throughout the entire production cycle may not be the most biologically efficient or economically optimal feeding strategy. In addition, data regarding the effects of ternary emulsifier blends on nutrient utilization, gastrointestinal function, and carcass characteristics in broilers fed reduced-energy diets remain scarce. Consequently, there is a need to evaluate whether a phase-specific supplementation approach can maximize the benefits of emulsifier supplementation while improving cost-effectiveness under practical commercial production conditions.

Therefore, this study was conducted to evaluate the effects of a ternary emulsifier blend comprising sorbitan esters, lysolecithin, and de-oiled lecithin on growth performance, nutrient utilization, gastrointestinal retention time, and carcass characteristics in broilers fed reduced-energy diets. In addition, the study investigated a phase-specific supplementation strategy in which higher emulsifier inclusion levels were provided during the starter and grower phases and reduced during the finisher phase. The objective was to determine whether this adaptive feeding approach could partially compensate for reduced dietary energy, improve nutrient utilization efficiency, and maintain productive performance while offering a practical and economically sustainable strategy for commercial broiler production.

## MATERIALS AND METHODS

### Ethical approval

All experimental procedures involving broiler chickens were reviewed and approved by the Kasetsart University Institutional Animal Care and Use Committee, Bangkok, Thailand (Approval No. ACKU65-AGR-011). The study was conducted in accordance with the institutional guidelines for the care and use of animals in research and the principles of Good Agricultural Practices for broiler production. Before the experiment, all birds were inspected for health status and managed under controlled environmental conditions with appropriate stocking density, ventilation, temperature, lighting, vaccination, and *ad libitum* access to feed and water. Throughout the 35-day experimental period, birds were monitored daily for general health, behavior, morbidity, mortality, and signs of stress. Handling was minimized and performed by trained personnel to reduce discomfort. Birds selected for carcass evaluation were humanely euthanized by carbon dioxide inhalation in a controlled chamber, followed by exsanguination, ensuring rapid loss of consciousness and minimizing pain and distress. All efforts were made to safeguard animal welfare and use the minimum number of birds required to obtain reliable scientific data.

### Study period and location

The study was conducted from January to April 2024 at the Poultry Research Center Farm, Department of Animal Science, Faculty of Agriculture, Kasetsart University, Bangkok, Thailand.

### Study design, experimental animals, and dietary treatments

A total of 750 one-day-old Ross 308 male broiler chicks were randomly allocated to five dietary treatments in a completely randomized design. Each treatment consisted of six replicates, with 25 birds per replicate pen.

**Experimental diets were formulated for three feeding phases:** starter (1–10 days), grower (11–24 days), and finisher (25–35 days). The dietary treatments consisted of: (T1) PC with standard ME; (T2) NC with ME reduced by 150 kcal/kg relative to the PC; (T3) NC supplemented with 250 g/ton emulsifier; (T4) NC supplemented with 200 g/ton emulsifier; and (T5) NC supplemented with 250 g/ton emulsifier during the starter and grower phases followed by 200 g/ton during the finisher phase. The ingredients and chemical composition of the experimental diets are presented in Table 1.

The PC diets were formulated to meet standard ME requirements for broilers, whereas the NC diets were formulated with a reduction of 150 kcal/kg ME. The calculated ME values for the PC and NC diets were 2,975 and 2,825 kcal/kg during the starter phase, 3,050 and 2,900 kcal/kg during the grower phase, and 3,100 and 2,950 kcal/kg during the finisher phase, respectively. The reduction in dietary energy was primarily achieved by decreasing the inclusion of energy-dense ingredients, such as corn, and increasing the inclusion of rice bran, thereby resulting in higher dietary fiber concentrations. The inclusion level of added fat remained relatively constant among treatments. Crude protein (CP) and essential amino acid concentrations (lysine, methionine, threonine, and valine) were maintained at similar levels across the PC and NC diets to minimize confounding effects of nutrient imbalance.

The nutrient values presented in Table 1 were calculated values. Analyzed nutrient composition and fatty acid profiles of the dietary fat source were not determined in the present study. The increased fiber concentration and reduced energy density of the NC diets were expected to negatively affect lipid digestion efficiency and therefore provided an appropriate nutritional model for evaluating the efficacy of emulsifier supplementation.

Birds were reared in floor pens within a controlled-environment poultry house. Each replicate pen contained 25 birds and provided 1.8 m² of floor space, corresponding to a stocking density of 13.89 birds/m². The poultry house was equipped with an evaporative cooling system, programmable artificial lighting, automated electric heating, and tunnel ventilation. The brooding temperature was initially maintained at 34°C and gradually reduced to 28°C during the first 3 weeks of the experiment. The lighting schedule consisted of 18 h light and 6 h darkness per 24-h cycle throughout days 10–35 of the experimental period. Feed and water were provided ad libitum, and birds were vaccinated in accordance with standard commercial management practices.

**Table 1:** Ingredients and calculated nutrient composition of experimental diets (% on an as-fed basis).

**Items**	**S-PC**	**S-NC**	**G-PC**	**G-NC**	**F-PC**	**F-NC**
Ingredients						
Corn	52.76	42.95	58.17	48.36	62.54	52.74
Palm oil	2.50	2.50	2.50	2.50	2.50	2.50
Rice solvent bran	5.92	17.92	4.86	16.87	6.32	18.33
Soybean meal (48% CP)	34.93	32.76	31.55	29.38	26.11	23.94
L-Lysine	0.28	0.30	0.21	0.23	0.25	0.27
DL-Methionine	0.38	0.38	0.33	0.33	0.31	0.31
L-Threonine	0.11	0.12	0.06	0.08	0.06	0.08
L-Valine	0.07	0.07	0.04	0.04	0.06	0.05
Monodicalcium phosphate	1.11	1.02	0.67	0.57	0.33	0.24
Calcium carbonate	1.18	1.21	0.86	0.90	0.78	0.81
Salt	0.30	0.30	0.30	0.30	0.30	0.30
Phytase + NSP enzyme (100 g/t; −70 kcal/kg)	0.01	0.01	0.01	0.01	0.01	0.01
Choline chloride (60%)	0.28	0.28	0.27	0.27	0.25	0.25
Premix	0.18	0.18	0.18	0.18	0.18	0.18
Total	100.00	100.00	100.00	100.00	100.00	100.00
Chemical composition						
ME for poultry (kcal/kg)*	2,975	2,825	3,050	2,900	3,100	2,950
Protein (%)	23.00	23.00	21.50	21.50	19.50	19.50
Fat (%)	4.76	4.50	4.91	4.65	5.03	4.77
Fiber (%)	4.12	5.19	3.93	5.00	3.89	4.95
Calcium (%)	0.95	0.95	0.75	0.75	0.65	0.65
Total phosphorus (%)	0.88	1.00	0.76	0.88	0.69	0.81
Available phosphorus (%)	0.50	0.50	0.42	0.42	0.36	0.36
Salt (%)	0.34	0.34	0.33	0.33	0.32	0.32
Lysine (%)	1.43	1.44	1.28	1.29	1.17	1.18
Methionine + cystine (%)	1.08	1.08	0.99	0.99	0.93	0.93
Methionine (%)	0.70	0.71	0.64	0.64	0.60	0.61
Threonine (%)	0.98	0.98	0.88	0.88	0.80	0.80
Tryptophan (%)	0.28	0.27	0.26	0.25	0.23	0.22
Valine (%)	1.14	1.15	1.04	1.05	0.96	0.97
Choline (mg/kg)	1,700	1,700	1,600	1,600	1,500	1,500
Lysine digestibility (%)	1.32	1.32	1.18	1.18	1.08	1.08
Methionine digestibility (%)	0.68	0.68	0.62	0.62	0.58	0.58
Methionine + cystine digestibility (%)	1.00	1.00	0.92	0.92	0.86	0.86
Threonine digestibility (%)	0.88	0.88	0.79	0.79	0.72	0.72
Tryptophan digestibility (%)	0.26	0.26	0.24	0.24	0.21	0.21
Valine digestibility (%)	1.00	1.00	0.91	0.91	0.84	0.84

S = Starter; G = Grower; F = Finisher; PC = Positive control; NC = Negative control; ME = Metabolizable energy; NSP = Non-starch polysaccharide.

The reduction in dietary ME in NC diets was achieved mainly through adjustments in corn and rice solvent bran inclusion levels, resulting in increased fiber and reduced-energy density while maintaining similar protein and amino acid concentrations.

### Emulsifier product and specification

The emulsifier evaluated in this study was a commercial soy lysolecithin-based product, BergaFit 60 SO Lyso GMO (Berg+Schmidt Asia Pte. Ltd., Singapore, Singapore), formulated for use in feeds for poultry, swine, ruminants, aquaculture species, and companion animals. According to the manufacturer's specifications, the product contains soy lysolecithin on a carrier with acetone-insoluble matter ≥34%, ash ≤40%, and an acid value ≤42 mg KOH/g.

The emulsifier consisted of a blend of sorbitan esters, lysolecithin, and de-oiled lecithin and was incorporated into experimental diets at 250 g/ton, 200 g/ton, or under a phase-specific step-down supplementation strategy as described previously.

### Productive performance and carcass yield

Individual body weight (BW) was recorded on day 1 and at the end of each feeding phase (days 10, 24, and 35). Feed consumption was recorded on a pen basis at the end of each respective growth phase.

Productive performance variables, including BW, average daily gain (ADG), FI, FCR, corrected FCR (cFCR), feed cost per gain (FCG), and mortality, were evaluated during four periods: starter phase (1–10 days), grower phase (11–24 days), finisher phase (25–35 days), and the overall experimental period (1–35 days) [18, 19]. The cFCR was calculated by adjusting FCR for mortality.

At 35 days of age, two birds per replicate were randomly selected for carcass evaluation. Selection was restricted to birds with BW values close to the treatment mean. Live BW was recorded before slaughter. Birds were humanely euthanized by carbon dioxide (CO₂) inhalation in a controlled chamber followed by exsanguination. Carcasses were defeathered and eviscerated, and internal organs were removed to obtain eviscerated carcass weight. Abdominal fat was excised and weighed separately.

The eviscerated carcasses were subsequently dissected into commercial cuts, including wings, inner breast (Pectoralis minor), outer breast (*Pectoralis major*), thighs, and drumsticks. Individual carcass components were weighed separately. Carcass yield was calculated as the percentage of eviscerated carcass weight relative to live BW. The relative weights of wings, inner breast, outer breast, thighs, drumsticks, and abdominal fat were expressed as percentages of eviscerated carcass weight [20].

### Feed digestibility, AME, and fecal score evaluation

On day 30, two birds per replicate (12 birds per treatment) were selected for digestibility assessment. Birds were transferred to metabolic cages (two birds per cage) and fed experimental diets containing 0.5% chromium oxide (Cr₂O₃) and 1% Celite as external markers.

The interval between marker administration and the appearance of green-colored feces was recorded as gastrointestinal retention time. Excreta were partially collected over a 6-h period for subsequent analyses [21]. Fecal consistency was visually scored using a 4-point scale, where 0 = normal, 1 = slightly wet, 2 = wet, and 3 = very wet [22].

A 100-g subsample of fresh excreta was weighed immediately and dried to determine dry matter content. Dried excreta samples were ground through a 1-mm screen, whereas feed samples were ground through a 0.5-mm screen before laboratory analyses.

Feed and excreta samples were analyzed for OM using standard proximate analytical procedures based on moisture and ash determination. AME corrected for nitrogen was determined by analyzing GE using an adiabatic oxygen bomb calorimeter and nitrogen concentration using the Kjeldahl method [23].

AME was calculated using the following equation:

AME = GEdiet − (GEexcreta × (Markerdiet/Markerexcreta))

where GE represents gross energy. Marker concentrations in the diet and excreta were used to quantify digesta flow and nutrient retention, with Cr₂O₃ as an indigestible marker.

### Statistical analysis

Data were analyzed using a one-way analysis of variance using SAS software [24]. When significant treatment effects were detected, means were separated using Duncan's multiple range test. Statistical significance was declared at p < 0.05. Results are presented as mean ± standard deviation.

The experimental unit was the replicate pen. The sample size (n = 6 replicates per treatment) was considered sufficient to detect biologically meaningful differences in broiler growth performance based on previous studies conducted under similar experimental conditions.

## RESULTS

### Growth performance during the starter phase (1–10 days)

The nutrient composition of the experimental diets is presented in Table 1. The diets were categorized into PC and NC groups. The PC diets contained 150 kcal/kg more ME than the NC diets. In addition, the PC diets contained slightly higher fat concentrations and lower fiber concentrations than the NC diets.

The effects of emulsifier supplementation on broiler performance during the starter phase (1–10 days) are presented in Table 2. Initial BW did not differ among treatments (p > 0.05), indicating satisfactory flock uniformity. Final BW was significantly affected by dietary treatment (p = 0.02). The highest final BW was observed in T1 (339.30 ± 29.44 g), which did not differ from T5 (334.07 ± 28.92 g) but was significantly greater than T2 (324.61 ± 41.98 g). Treatments T3 (331.93 ± 33.40 g) and T4 (330.06 ± 43.80 g) showed intermediate values and did not differ significantly from either T1 or T2.

No significant differences were observed among treatments for ADG and FI (p = 0.10 and p = 0.89, respectively). However, FCR and cFCR were significantly influenced by dietary treatment (p < 0.01). The most favorable FCR and cFCR values were recorded in T1 (1.021 ± 0.01), followed by T5 (1.043 ± 0.00), T4 (1.043 ± 0.01), and T3 (1.046 ± 0.02), all of which were significantly improved compared with T2 (1.072 ± 0.02). FCG was not significantly affected by treatment (p = 0.07), ranging from 18.121 ± 0.36 to 18.576 ± 0.28. No mortality was observed in any treatment during the starter phase.

**Table 2:** Effects of emulsifier supplementation on growth performance of broilers during the starter phase (1–10 days).

**Items**	**T1**	**T2**	**T3**	**T4**	**T5**	**p-value**	**SEM**
Initial BW (g)	40.60 ± 3.21	40.14 ± 3.21	40.43 ± 3.04	40.12 ± 3.06	40.14 ± 3.04	0.62	0.12
Uniformity (%)	92.05 ± 0.61	91.89 ± 0.46	92.44 ± 0.97	92.37 ± 0.35	92.44 ± 0.50	0.49	0.12
BW (g)	339.30 ± 29.44ᵃ	324.61 ± 41.98ᵇ	331.93 ± 33.40ᵃᵇ	330.06 ± 43.80ᵃᵇ	334.07 ± 28.92ᵃ	0.02	1.53
ADG (g/day)	29.87 ± 0.66	28.44 ± 1.13	29.15 ± 0.72	29.00 ± 0.82	29.39 ± 0.60	0.10	0.16
FI (g/day)	30.50 ± 0.31	30.48 ± 0.97	30.49 ± 1.09	30.22 ± 0.36	30.66 ± 0.57	0.89	0.13
FCR	1.021 ± 0.01ᶜ	1.072 ± 0.02ᵃ	1.046 ± 0.02ᵇ	1.043 ± 0.02ᵇ	1.043 ± 0.00ᵇ	0.001	0.004
cFCR	1.021 ± 0.01ᶜ	1.072 ± 0.02ᵃ	1.046 ± 0.02ᵇ	1.043 ± 0.02ᵇ	1.043 ± 0.00ᵇ	0.001	0.004
FCG	18.180 ± 0.26	18.576 ± 0.28	18.174 ± 0.31	18.121 ± 0.36	18.130 ± 0.08	0.07	0.06
Mortality (%)	0.00 ± 0.00	0.00 ± 0.00	0.00 ± 0.00	0.00 ± 0.00	0.00 ± 0.00	–	–

A–CValues within the same row bearing different superscripts differ significantly (p < 0.01).

a,bValues within the same row bearing different superscripts differ significantly (p < 0.05).

SEM = Standard error of the mean.

BW = Body weight; ADG = Average daily gain; FI = Feed intake; FCR = Feed conversion ratio; cFCR = Corrected feed conversion ratio; FCG = Feed cost per gain; T1 = Positive control; T2 = Negative control (150 kcal/kg lower metabolizable energy than T1); T3 = Negative control + 250 g/ton emulsifier; T4 = Negative control + 200 g/ton emulsifier; T5 = Negative control + 250 g/ton emulsifier during the starter and grower phases and 200 g/ton during the finisher phase.

The negative control diets were formulated to contain 150 kcal/kg lower ME than the positive control diets, mainly by reducing corn and increasing rice solvent bran inclusion. Nutrient values are calculated values.

### Growth performance during the grower phase (11–24 days)

The effects of dietary emulsifier supplementation on broiler performance during the grower phase are presented in Table 3. Final BW at day 24 was significantly influenced by dietary treatment (p = 0.04). Birds receiving T3 (1,434.93 ± 145.15 g), T4 (1,425.89 ± 139.34 g), and T5 (1,423.61 ± 132.64 g) exhibited greater BW than birds receiving T2 (1,386.32 ± 148.31 g). The BW of birds in T1 (1,407.35 ± 146.32 g) did not differ significantly from either T2 or the emulsifier-supplemented treatments.

No significant differences were observed among treatments for ADG, FI, FCR, cFCR, or FCG (p > 0.05).

### Cumulative growth performance during the starter and grower phases (1–24 days)

The cumulative performance of broilers from day 1 to day 24 is presented in Table 4. No significant differences were detected among treatments for ADG, FI, FCR, cFCR, or FCG (p > 0.05). Mortality remained low across all treatments, ranging from 0.67 ± 1.63% to 2.00 ± 4.90%, with no significant differences among treatment groups (p > 0.05).

### Growth performance during the finisher phase (25–35 days)

The effects of dietary emulsifier supplementation on broiler performance during the finisher phase are presented in Table 5. Final BW at day 35 was not significantly affected by treatment (p = 0.15). Likewise, no significant differences were observed for ADG, FI, FCR, cFCR, FCG, or mortality (p > 0.05).

### Cumulative growth performance during the grower and finisher phases (11–35 days)

The cumulative performance of broilers from day 11 to day 35 is presented in Table 6. Final BW, ADG, FI, FCR, cFCR, FCG, and mortality were not significantly affected by dietary treatment (p > 0.05). Mortality remained low, ranging from 0.00 ± 0.00% to 0.83 ± 1.86%, with no significant differences among treatments (p > 0.05).

**Table 3:** Effects of emulsifier supplementation on growth performance of broilers during the grower phase (11–24 days).

**Items**	**T1**	**T2**	**T3**	**T4**	**T5**	**p-value**	**SEM**
Initial BW (g)	339.30 ± 29.44ᵃ	324.61 ± 41.98ᵇ	331.93 ± 33.40ᵃᵇ	330.06 ± 43.80ᵃᵇ	334.07 ± 28.92ᵃ	0.02	1.53
BW (g)	1,407.35 ± 146.32ᵃᵇ	1,386.32 ± 148.31ᵇ	1,434.93 ± 145.15ᵃ	1,425.89 ± 139.34ᵃ	1,423.61 ± 132.64ᵃ	0.04	6.39
ADG (g/day)	76.50 ± 3.85	75.69 ± 2.18	78.80 ± 4.32	78.21 ± 3.47	77.82 ± 2.57	0.56	0.63
FI (g/day)	98.37 ± 3.94	99.35 ± 2.32	99.67 ± 5.30	101.07 ± 3.54	99.65 ± 2.48	0.82	0.67
FCR	1.287 ± 0.04	1.313 ± 0.02	1.265 ± 0.02	1.293 ± 0.03	1.281 ± 0.04	0.20	0.006
cFCR	1.287 ± 0.04	1.324 ± 0.03	1.274 ± 0.03	1.323 ± 0.08	1.290 ± 0.04	0.33	0.009
FCG	22.224 ± 0.74	22.253 ± 0.51	21.490 ± 0.43	22.300 ± 1.37	21.763 ± 0.64	0.38	0.16
Mortality (%)	0.00 ± 0.00	0.67 ± 1.63	0.67 ± 1.63	2.00 ± 4.90	0.67 ± 1.63	0.78	0.48

^a,b^Values within the same row bearing different superscripts differ significantly (p < 0.05).

SEM = Standard error of the mean.

BW = Body weight; ADG = Average daily gain; FI = Feed intake; FCR = Feed conversion ratio; cFCR = Corrected feed conversion ratio; FCG = Feed cost per gain; T1 = Positive control; T2 = Negative control (150 kcal/kg lower metabolizable energy than T1); T3 = Negative control + 250 g/ton emulsifier; T4 = Negative control + 200 g/ton emulsifier; T5 = Negative control + 250 g/ton emulsifier during the starter and grower phases and 200 g/ton during the finisher phase.

Note: The BW value for T1 appears as 1,407.35 ± 1463.32 in the manuscript. This is likely a typographical error and should probably be verified against the original dataset (possibly *146.32* rather than 1463.32).

**Table 4:** Effects of emulsifier supplementation on cumulative growth performance of broilers during the starter and grower phases (1–24 days).

**Items**	**T1**	**T2**	**T3**	**T4**	**T5**	**p-value**	**SEM**
Initial BW (g)	40.60 ± 3.21	40.14 ± 3.21	40.43 ± 3.04	40.12 ± 3.06	40.14 ± 3.04	0.62	0.12
BW (g)	1,407.35 ± 1463.32ᵃᵇ	1,386.32 ± 148.31ᵇ	1,434.93 ± 145.15ᵃ	1,425.89 ± 139.34ᵃ	1,423.61 ± 132.64ᵃ	0.04	6.39
ADG (g/day)	57.07 ± 2.49	56.00 ± 1.11	58.11 ± 2.77	57.70 ± 2.12	57.64 ± 1.69	0.55	0.40
FI (g/day)	70.09 ± 2.38	70.65 ± 1.35	70.84 ± 3.53	71.55 ± 2.16	70.90 ± 1.42	0.89	0.42
FCR	1.229 ± 0.04	1.262 ± 0.02	1.219 ± 0.01	1.241 ± 0.03	1.231 ± 0.03	0.10	0.005
cFCR	1.229 ± 0.03	1.272 ± 0.03	1.228 ± 0.03	1.289 ± 0.07	1.239 ± 0.03	0.28	0.008
FCG	21.341 ± 0.60	21.505 ± 0.48	20.824 ± 0.44	21.505 ± 1.25	21.021 ± 0.54	0.44	0.14
Mortality (%)	0.00 ± 0.00	0.80 ± 1.79	0.67 ± 1.63	2.00 ± 4.90	0.67 ± 1.63	0.78	0.48

^a,b^Values within the same row bearing different superscripts differ significantly (p < 0.05).

SEM = Standard error of the mean.

BW = Body weight; ADG = Average daily gain; FI = Feed intake; FCR = Feed conversion ratio; cFCR = Corrected feed conversion ratio; FCG = Feed cost per gain; T1 = Positive control; T2 = Negative control (150 kcal/kg lower metabolizable energy than T1); T3 = Negative control + 250 g/ton emulsifier; T4 = Negative control + 200 g/ton emulsifier; T5 = Negative control + 250 g/ton emulsifier during the starter and grower phases and 200 g/ton during the finisher phase.

Note: The BW value for T1 is reported as 1,407.35 ± 1463.32 in the source table and should be verified against the original dataset because the standard deviation appears unusually large.

**Table 5:** Effects of emulsifier supplementation on growth performance of broilers during the finisher phase (25–35 days).

**Items**	**T1**	**T2**	**T3**	**T4**	**T5**	**p-value**	**SEM**
Initial BW (g)	1,407.35 ± 1463.32ᵃᵇ	1,386.32 ± 148.31ᵇ	1,434.93 ± 145.15ᵃ	1,425.89 ± 139.34ᵃ	1,423.61 ± 132.64ᵃ	0.04	6.39
BW (g)	2,468.16 ± 314.30	2,427.15 ± 272.17	2,474.15 ± 283.13	2,497.91 ± 314.58	2,487.38 ± 256.22	0.15	10.41
ADG (g/day)	98.52 ± 4.63	94.21 ± 3.89	94.62 ± 8.46	97.48 ± 5.76	96.67 ± 7.57	0.78	1.17
FI (g/day)	162.81 ± 7.55	165.54 ± 7.42	166.78 ± 10.43	167.37 ± 8.86	165.21 ± 6.93	0.91	1.50
FCR	1.655 ± 0.10	1.757 ± 0.05	1.767 ± 0.08	1.720 ± 0.11	1.715 ± 0.10	0.33	0.02
cFCR	1.655 ± 0.10	1.772 ± 0.05	1.767 ± 0.08	1.720 ± 0.11	1.726 ± 0.10	0.28	0.02
FCG	27.593 ± 1.68	28.734 ± 0.77	28.754 ± 1.34	27.968 ± 1.74	28.067 ± 1.64	0.65	0.27
Mortality (%)	0.00 ± 0.00	0.83 ± 1.86	0.00 ± 0.00	0.00 ± 0.00	0.67 ± 1.63	0.54	0.20

^a,b^Values within the same row bearing different superscripts differ significantly (p < 0.05).

SEM = Standard error of the mean.

BW = Body weight; ADG = Average daily gain; FI = Feed intake; FCR = Feed conversion ratio; cFCR = Corrected feed conversion ratio; FCG = Feed cost per gain; T1 = Positive control; T2 = Negative control (150 kcal/kg lower metabolizable energy than T1); T3 = Negative control + 250 g/ton emulsifier; T4 = Negative control + 200 g/ton emulsifier; T5 = Negative control + 250 g/ton emulsifier during the starter and grower phases and 200 g/ton during the finisher phase.

**Table 6:** Effects of emulsifier supplementation on cumulative growth performance of broilers during the grower and finisher phases (11–35 days).

**Items**	**T1**	**T2**	**T3**	**T4**	**T5**	**p-value**	**SEM**
Initial BW (g)	339.30 ± 29.44ᵃ	324.61 ± 41.98ᵇ	331.93 ± 33.40ᵃᵇ	330.06 ± 43.80ᵃᵇ	334.07 ± 28.92ᵃ	0.02	1.53
BW (g)	2,468.16 ± 314.30	2,427.15 ± 272.17	2,474.15 ± 283.13	2,497.91 ± 314.58	2,487.38 ± 256.22	0.15	10.41
ADG (g/day)	86.19 ± 3.04	83.83 ± 2.31	85.76 ± 5.95	86.69 ± 4.22	86.12 ± 4.45	0.85	0.77
FI (g/day)	126.73 ± 5.12	128.47 ± 3.88	129.19 ± 7.49	130.25 ± 5.65	128.50 ± 4.30	0.88	0.99
FCR	1.471 ± 0.06	1.532 ± 0.02	1.508 ± 0.04	1.504 ± 0.06	1.494 ± 0.05	0.36	0.009
cFCR	1.471 ± 0.06	1.559 ± 0.05	1.518 ± 0.04	1.538 ± 0.09	1.515 ± 0.07	0.30	0.01
FCG	24.904 ± 0.94	25.682 ± 0.82	25.094 ± 0.60	25.399 ± 1.56	25.031 ± 1.11	0.77	0.19
Mortality (%)	0.00 ± 0.00	1.63 ± 3.65	0.67 ± 1.63	2.00 ± 4.90	1.33 ± 2.07	0.82	0.54

^a,b^Values within the same row bearing different superscripts differ significantly (p < 0.05).

SEM = Standard error of the mean.

BW = Body weight; ADG = Average daily gain; FI = Feed intake; FCR = Feed conversion ratio; cFCR = Corrected feed conversion ratio; FCG = Feed cost per gain; T1 = Positive control; T2 = Negative control (150 kcal/kg lower metabolizable energy than T1); T3 = Negative control + 250 g/ton emulsifier; T4 = Negative control + 200 g/ton emulsifier; T5 = Negative control + 250 g/ton emulsifier during the starter and grower phases and 200 g/ton during the finisher phase.

### Overall growth performance (1–35 days)

The cumulative performance of broilers throughout the entire experimental period is presented in Table 7. No significant differences were observed among treatments for ADG, which ranged from 68.01 ± 1.43 g/day in T2 to 70.20 ± 3.06 g/day in T4 (p = 0.79). FI was also comparable among treatments (p = 0.90), ranging from 99.24 ± 3.73 to 101.67 ± 4.10 g/day.

Similarly, FCR and cFCR were not significantly affected by treatment (p = 0.22 and p = 0.23, respectively). FCG ranged from 24.084 ± 0.82 in T1 to 24.871 ± 0.84 in T2 and did not differ significantly among treatments (p = 0.71). Mortality remained low across all treatment groups and did not differ significantly (p = 0.82).

**Table 7:** Effects of emulsifier supplementation on overall growth performance of broilers during the experimental period (1–35 days).

**Items**	**T1**	**T2**	**T3**	**T4**	**T5**	**p-value**	**SEM**
Initial BW (g)	40.60 ± 3.21	40.14 ± 3.21	40.43 ± 3.04	40.12 ± 3.06	40.14 ± 3.04	0.62	0.12
BW (g)	2,468.16 ± 314.30	2,427.15 ± 272.17	2,474.15 ± 283.13	2,497.91 ± 314.58	2,487.38 ± 256.22	0.15	10.41
ADG (g/day)	70.10 ± 2.32	68.01 ± 1.43	69.58 ± 4.40	70.20 ± 3.06	69.91 ± 3.30	0.79	0.57
FI (g/day)	99.24 ± 3.73	100.48 ± 2.58	100.99 ± 5.64	101.67 ± 4.10	100.54 ± 3.09	0.90	0.72
FCR	1.416 ± 0.05	1.477 ± 0.02	1.452 ± 0.03	1.449 ± 0.05	1.439 ± 0.04	0.22	0.008
cFCR	1.416 ± 0.05	1.503 ± 0.05	1.462 ± 0.03	1.482 ± 0.09	1.460 ± 0.06	0.23	0.01
FCG	24.084 ± 0.82	24.871 ± 0.84	24.271 ± 0.57	24.577 ± 1.44	24.224 ± 1.00	0.71	0.18
Mortality (%)	0.00 ± 0.00	1.63 ± 3.65	0.67 ± 1.63	2.00 ± 4.90	1.33 ± 2.07	0.82	0.54

SEM = Standard error of the mean.

BW = Body weight; ADG = Average daily gain; FI = Feed intake; FCR = Feed conversion ratio; cFCR = Corrected feed conversion ratio; FCG = Feed cost per gain; T1 = Positive control; T2 = Negative control (150 kcal/kg lower metabolizable energy than T1); T3 = Negative control + 250 g/ton emulsifier; T4 = Negative control + 200 g/ton emulsifier; T5 = Negative control + 250 g/ton emulsifier during the starter and grower phases and 200 g/ton during the finisher phase.

### Feed digestibility, AME, fecal characteristics, and gastrointestinal retention time

The effects of dietary emulsifier supplementation on fecal characteristics, gastrointestinal retention time, feed digestibility, and AME at 30 days of age are presented in Table 8.

Fecal scores did not differ among treatments (p = 0.86), with values ranging from 0.70 ± 1.10 in T2 to 1.42 ± 1.24 in T5, indicating generally normal to slightly wet feces across treatments.

Mean gastrointestinal retention time was significantly influenced by dietary treatment (p = 0.01). The longest retention time was observed in T1 (199.80 ± 31.25 min), which was significantly greater than those observed in T2 (162.20 ± 24.30 min), T4 (152.00 ± 18.56 min), and T5 (153.50 ± 11.61 min). Treatment T3 (180.67 ± 27.35 min) showed intermediate values and did not differ significantly from the other treatments.

No significant differences were observed among treatments for total tract digestibility (p = 0.73) or OM digestibility (p = 0.80). AME tended to be numerically greater in emulsifier-supplemented groups (3,085–3,111 kcal/kg) than in the NC group (2,951 kcal/kg); however, these differences were not statistically significant (p = 0.69).

### Carcass characteristics

The effects of dietary emulsifier supplementation on carcass characteristics at 35 days of age are presented in Table 9. Carcass yield tended to differ among treatments (p = 0.053), with T1 (85.81 ± 1.23%) showing the highest numerical value and T5 (84.85 ± 1.68%) showing an intermediate value.

**Table 8:** Effects of emulsifier supplementation on fecal characteristics, gastrointestinal retention time, digestibility, and AME of broilers at 30 days of age.

**Items**	**T1**	**T2**	**T3**	**T4**	**T5**	**p-value**	**SEM**
Fecal score (0–3)*	0.90 ± 1.34	0.70 ± 1.10	0.92 ± 0.92	1.08 ± 1.02	1.42 ± 1.24	0.86	0.20
Mean retention time (min)	199.80 ± 31.25ᵃ	162.20 ± 24.30ᵇ	180.67 ± 27.35ᵃᵇ	152.00 ± 18.56ᵇ	153.50 ± 11.61ᵇ	0.01	5.32
Total digestibility (%)	83.17 ± 4.69	78.46 ± 8.93	83.35 ± 4.64	82.23 ± 6.10	82.77 ± 6.38	0.73	1.17
OM digestibility (%)	85.26 ± 4.66	80.75 ± 9.24	84.62 ± 4.31	84.26 ± 5.64	84.39 ± 6.06	0.80	1.14
AME (kcal/kg)	3,117.68 ± 102.75	2,951.05 ± 331.29	3,085.46 ± 150.91	3,111.24 ± 196.46	3,109.18 ± 210.73	0.69	39.38

^a,b^Values within the same row bearing different superscripts differ significantly (p < 0.05).

SEM = Standard error of the mean.

T1 = Positive control; T2 = Negative control (150 kcal/kg lower metabolizable energy than T1); T3 = Negative control + 250 g/ton emulsifier; T4 = Negative control + 200 g/ton emulsifier; T5 = Negative control + 250 g/ton emulsifier during the starter and grower phases and 200 g/ton during the finisher phase; AME = Apparent metabolizable energy; OM = Organic matter.

Fecal score: 0 = Normal; 1 = Slightly wet; 2 = Wet; 3 = Very wet.

However, no significant differences were observed among treatments for wing, inner breast, outer breast, drumstick, thigh, or abdominal fat percentages (p > 0.05).

**Table 9:** Effects of emulsifier supplementation on carcass characteristics of broilers at 35 days of age.

**Items**	**T1**	**T2**	**T3**	**T4**	**T5**	**p-value**	**SEM**
Carcass yield (%)	85.81 ± 1.23	84.28 ± 1.75	84.03 ± 1.13	84.08 ± 1.73	84.85 ± 1.68	0.053	0.22
Wing (%)	8.40 ± 1.37	8.68 ± 0.49	8.91 ± 0.47	8.94 ± 0.66	9.03 ± 0.67	0.35	0.10
Inner breast (%)	5.29 ± 1.58	5.03 ± 0.19	5.09 ± 0.41	4.80 ± 0.43	5.01 ± 0.63	0.70	0.10
Outer breast (%)	26.14 ± 1.61	27.20 ± 1.62	27.01 ± 1.14	26.69 ± 1.11	26.78 ± 1.17	0.45	0.18
Drumstick (%)	11.71 ± 0.83	11.95 ± 0.64	11.99 ± 0.41	11.86 ± 1.06	12.03 ± 0.59	0.86	0.10
Thigh (%)	16.05 ± 1.24	15.81 ± 0.76	15.37 ± 1.04	15.23 ± 1.23	15.16 ± 0.89	0.23	0.14
Abdominal fat (%)	1.18 ± 0.19	1.13 ± 0.25	1.19 ± 0.35	1.07 ± 0.28	1.16 ± 0.28	0.86	0.04

SEM = Standard error of the mean.

T1 = Positive control; T2 = Negative control (150 kcal/kg lower metabolizable energy than T1); T3 = Negative control + 250 g/ton emulsifier; T4 = Negative control + 200 g/ton emulsifier; T5 = Negative control + 250 g/ton emulsifier during the starter and grower phases and 200 g/ton during the finisher phase.

## DISCUSSION

### Effects of emulsifier supplementation on growth performance during the starter phase

The present study investigated the effects of emulsifier supplementation at different dietary inclusion levels on the performance of broilers during the starter phase (1–10 days). The improvements in BW and feed efficiency observed during this phase suggest enhanced nutrient utilization, particularly of dietary lipids. These findings are consistent with previous studies demonstrating that emulsifier supplementation improves fat digestion and energy availability in young broilers with immature digestive systems. The greater response observed during early life may be explained by limited endogenous bile secretion and lipase activity at this developmental stage. The numerical improvement in digestibility observed in the present study may be attributable to enhanced fat digestion, which agrees with previous reports [5].

Emulsifier supplementation has been reported to improve FI and BW in broilers during the starter phase. Furthermore, the inclusion of emulsifiers has been associated with improved FCR, indicating more efficient conversion of feed into body mass, which is a critical factor in the economic production of broilers [25]. Emulsifiers improve lipid digestion by reducing the surface tension between dietary lipids and the aqueous phase of the digesta, thereby facilitating emulsification, micelle formation, and increasing the accessibility of lipids to digestive enzymes [26]. Consequently, dietary energy utilization is enhanced, particularly in energy-deficient diets such as T2, which contained 150 kcal/kg less ME than the PC diet.

The early-phase response observed in the present study may also be related to the complementary functions of the emulsifier components. Sorbitan esters act as non-ionic emulsifiers and may improve the dispersion of dietary lipid droplets, thereby increasing the surface area available for enzymatic hydrolysis. Lysolecithin, a lysophospholipid-rich component, may further facilitate micelle formation and lipid absorption, whereas de-oiled lecithin provides phospholipids that contribute to emulsion stability and lipid transport [26]. These complementary mechanisms may be particularly beneficial in young broilers whose endogenous lipid digestion capacity is not fully developed. The absence of treatment effects on FI further supports the hypothesis that improvements in growth performance were primarily attributable to enhanced nutrient absorption rather than increased feed consumption.

In addition, emulsifiers facilitate the digestion and absorption of dietary fats through several physiological and biochemical mechanisms that optimize nutrient utilization. Previous studies have also reported increased lipase activity following emulsifier supplementation, which enhances the hydrolysis of dietary fats into absorbable fatty acids and contributes to improved growth performance [5]. However, direct evidence regarding the efficacy of sorbitan esters in broiler nutrition remains limited. Therefore, the present findings should be considered preliminary evidence supporting the efficacy of this specific ternary emulsifier blend rather than confirmation of the independent contribution of each emulsifier component.

### Comparison with previous multi-component emulsifier systems

Previous investigations of multi-component emulsifier systems have primarily focused on combinations of lysolecithin, synthetic emulsifiers, and monoglycerides. For example, *Ghazalah et al.* [12] reported improvements in growth performance, intestinal morphology, and selected carcass traits in broilers fed low-energy diets supplemented with such combinations. In contrast, the present study evaluated a ternary blend containing sorbitan esters in addition to phospholipid-based emulsifiers, potentially providing an additional non-ionic emulsification mechanism. This distinction is important because responses to emulsifier supplementation are strongly influenced by emulsifier composition, dietary formulation, lipid source, and bird age.

### Effects of emulsifier supplementation during the grower and finisher phases

The greater BW observed during the grower phase in broilers receiving 200 and 250 g/ton emulsifier suggests improved nutrient utilization under reduced-energy conditions. This response may be attributed to enhanced lipid digestion and absorption, enabling the emulsifier to partially compensate for the dietary energy deficit and support growth performance.

In contrast, no significant treatment effects were observed during the finisher phase. This finding suggests that the response to emulsifier supplementation diminished as birds matured. The maturation of the digestive system, including increased endogenous secretion of bile salts and lipolytic enzymes, may reduce the dependence of older birds on exogenous emulsifiers [12]. Consequently, reducing emulsifier inclusion from 250 g/ton to 200 g/ton during the finisher phase did not adversely affect productive performance.

### Phase-dependent response to emulsifier supplementation

The absence of significant differences in overall performance from 1 to 35 days despite significant responses during the starter and grower phases may be explained by the progressive maturation of lipid digestion in broilers. As birds age, increased secretion of bile salts and pancreatic lipase enhances endogenous digestive capacity and reduces dependence on exogenous emulsifiers. Furthermore, compensatory growth during the later stages of production may have reduced differences among treatments that were evident earlier in life. Therefore, the lack of significant overall responses should not be interpreted as evidence of inefficacy but rather as an indication that the benefits of emulsifier supplementation are phase-dependent.

### Effects of emulsifiers on energy utilization

The use of emulsifiers in broiler diets has been shown to reduce dietary ME requirements while maintaining or enhancing productive performance. Previous studies have demonstrated that emulsifiers improve dietary fat digestibility, thereby allowing reductions in dietary energy without compromising growth performance [5]. The extent of energy reduction achievable depends on emulsifier type and dietary composition.

The inclusion of lecithin-based emulsifiers has previously allowed reductions in dietary ME while maintaining productive performance, suggesting that emulsifiers improve nutrient absorption and energy utilization efficiency [27]. Similarly, supplementation of emulsifiers in diets containing dietary oils reduced dietary ME requirements by 40 kcal/kg during days 1–21 and by 50 kcal/kg during days 22–49 while maintaining broiler performance during the early growth phase [28]. A meta-analysis further demonstrated that emulsifier supplementation increased weight gain by 1.62 g/day and reduced FCR by 0.04, confirming the effectiveness of emulsifiers in enhancing broiler performance under reduced-energy feeding programs [29].

### Functional roles of lysolecithin and de-oiled lecithin

Lysolecithin, a hydrolyzed derivative of lecithin, has been extensively studied and shown to improve FCR, BW gain, and total tract digestibility of energy and ether extract, particularly in reduced-energy diets [12, 30]. In addition, lysolecithin increases the production of beneficial short-chain fatty acids in the cecum, thereby contributing to intestinal health [31]. Furthermore, lysolecithin supplementation has been associated with improved litter quality and reduced incidence of footpad lesions, reflecting positive effects on bird welfare [11].

De-oiled lecithin exhibits strong emulsifying properties and contributes to improvements in growth performance, FCR, and carcass characteristics. Previous studies have reported increased breast muscle yield and reduced abdominal fat deposition in broilers fed de-oiled lecithin-supplemented diets [32]. De-oiled lecithin has also been associated with improvements in lipid metabolism, including increased high-density lipoprotein concentrations and reduced low-density lipoprotein concentrations. Moreover, improvements in intestinal morphology, including greater jejunal villus height and villus-to-crypt ratios, have been reported [33]. Reduced *E. coli* counts in excreta have also been observed, suggesting a beneficial role in maintaining gut microbial balance and intestinal health [32].

Although both lysolecithin and de-oiled lecithin have demonstrated substantial benefits, their efficacy may vary depending on dietary fat source, dietary energy concentration, and production objectives. Their benefits are generally more pronounced in reduced-energy diets, where enhanced nutrient utilization compensates for reduced-energy availability. However, under standard- or high-energy feeding conditions, their effects may be less evident. Therefore, practical application should consider production goals, economic factors, and physicochemical characteristics such as hydrophilic-lipophilic balance (HLB) and compatibility with dietary ingredients [10].

### Feed digestibility and AME responses

Although emulsifier supplementation numerically increased AME compared with the NC treatment, total tract digestibility and OM digestibility were not significantly affected. Therefore, the present results do not provide direct evidence that the emulsifier blend improved fat digestibility. Nevertheless, the numerical increase in AME may indicate partial improvement in energy utilization, which is consistent with the recognized role of emulsifiers in lipid dispersion and micelle formation. Future studies should include measurements of ether extract digestibility, ileal digestibility, and fatty acid digestibility to determine whether the observed increase in AME is directly associated with improved lipid utilization.

### Gastrointestinal retention time

Reducing dietary ME by 150 kcal/kg influenced gastrointestinal retention time, whereas supplementation with 200 g/ton emulsifier significantly reduced retention time. The longer retention time observed in the PC group may partly reflect its greater energy density and lower fiber concentration compared with the reduced-energy diets. In contrast, the NC diets contained higher levels of rice solvent bran and dietary fiber, which may have accelerated digesta passage.

The physiological mechanisms influencing retention time are closely associated with digestive efficiency, particularly lipid digestion and nutrient absorption. Improved nutrient digestibility, especially lipid utilization, may reduce retention time because nutrients are absorbed more rapidly and efficiently [34]. Emulsifiers can enhance dietary fat emulsification and absorption, thereby increasing digestive efficiency and potentially accelerating digesta passage through the gastrointestinal tract [3, 35]. However, this interpretation remains speculative because digesta passage kinetics and fat-specific digestibility were not directly evaluated.

The greater fiber concentration in the reduced-energy diets may also have contributed to the observed retention time responses. In the present study, the reduced-energy diets contained 1.06%–1.07% more fiber than the PC diet. High-fiber diets have been reported to decrease digesta retention time and increase passage rate through the gastrointestinal tract, which may reduce ME retention and dry matter metabolizability, particularly in diets containing fibrous ingredients such as wheat bran and oat hulls [36]. Therefore, the observed retention time response likely reflects the combined effects of dietary energy density, fiber concentration, and emulsifier supplementation rather than the effect of emulsifier supplementation alone.

### Fecal characteristics

Fecal scores did not differ significantly among treatments, indicating that reduced-energy diets and emulsifier supplementation did not adversely affect fecal consistency under the conditions of the present study. Although fecal score may indirectly reflect litter quality and bird welfare, litter moisture and footpad condition were not evaluated. Therefore, no definitive conclusions regarding welfare outcomes can be drawn from the present results.

### Carcass characteristics

Emulsifier supplementation in reduced-energy diets improved carcass yield to values comparable with those of the PC treatment without adversely affecting carcass component proportions or abdominal fat deposition. These findings suggest that emulsifiers can support muscle accretion and overall carcass development under reduced-energy feeding conditions.

The lower carcass yield observed in T2 (84.28%) compared with T1 (85.81%) is likely attributable to the reduction of 150 kcal/kg dietary ME, which may have limited growth potential and carcass deposition. In contrast, T5 (84.85%) exhibited numerically greater carcass yield than T2, suggesting that emulsifier supplementation partially restored carcass productivity. Previous studies have similarly reported increased carcass weight and carcass yield in broilers receiving emulsifier supplementation [12]. Improved energy utilization resulting from emulsifier supplementation may enhance protein and lipid metabolism, thereby supporting muscle development and increasing carcass yield [7]. Furthermore, emulsifiers may reduce fat excretion, resulting in more efficient utilization of dietary lipids and greater nutrient retention within muscle tissue rather than excreta [4].

### Practical implications of the study

The present study was conducted under tropical environmental conditions in Thailand, where elevated ambient temperature and humidity may negatively affect FI, nutrient utilization, and growth performance. Under these conditions, reducing dietary ME by 150 kcal/kg represented a practical nutritional challenge. The significant responses observed during the starter and grower phases suggest that the ternary emulsifier blend may partially support energy utilization in reduced-energy diets, particularly when young broilers possess limited digestive capacity. However, because these responses were not maintained through market age, the benefits of emulsifier supplementation should be interpreted as phase-dependent rather than continuous throughout the production cycle.

The practical relevance of the study is further strengthened by the use of palm oil and rice solvent bran, which are commonly used feed ingredients in Southeast Asian poultry production systems. The greater fiber concentration of the NC diet increased the challenge to nutrient utilization, whereas the inclusion of palm oil provided a commercially relevant model for evaluating emulsifier supplementation. Although the observed responses in gastrointestinal retention time and carcass yield suggest alterations in nutrient utilization, direct measurements of fatty acid digestibility, intestinal morphology, blood lipid profiles, and digestive enzyme activity are required to elucidate the underlying mechanisms. FCG was included as an economic indicator; however, no significant differences were observed among treatments. Consequently, the present study cannot conclusively demonstrate economic advantages. Nevertheless, the phase-specific supplementation strategy may offer opportunities for cost optimization by reducing emulsifier inclusion during the finisher phase, although this requires validation through commercial-scale economic analyses using current ingredient and additive prices.

## CONCLUSION

The present study demonstrated that supplementation of a ternary emulsifier blend containing sorbitan esters, lysolecithin, and de-oiled lecithin can partially compensate for a 150 kcal/kg reduction in dietary ME in broiler diets, particularly during the early stages of growth. Emulsifier supplementation improved BW, FCR, and cFCR during the starter phase and increased BW during the grower phase, indicating enhanced nutrient utilization under reduced-energy conditions. Although overall performance from 1 to 35 days was not significantly affected, emulsifier-supplemented treatments maintained productive performance comparable to that of birds receiving the standard-energy diet. In addition, emulsifier supplementation reduced gastrointestinal retention time and numerically increased AME, suggesting potential improvements in energy utilization efficiency. Carcass yield also tended to improve in emulsifier-supplemented groups, whereas carcass component proportions and abdominal fat deposition remained unaffected.

A major strength of this study was the evaluation of a novel ternary emulsifier system combined with a phase-specific supplementation strategy designed to match the changing digestive capacity of broilers throughout production. The findings indicate that higher emulsifier inclusion during the starter and grower phases followed by a reduced inclusion level during the finisher phase may represent a practical feeding strategy for maintaining broiler performance while reducing dietary energy density. This approach may be particularly relevant in commercial poultry production systems where feed cost reduction and efficient nutrient utilization are important economic objectives.

Several limitations should be acknowledged. Measurements of fat-specific digestibility, ileal nutrient digestibility, intestinal morphology, digestive enzyme activity, and blood lipid profiles were not included, limiting mechanistic interpretation of the observed responses. In addition, the digestibility assessment involved a relatively limited number of experimental units, and the economic evaluation was restricted to FCG without comprehensive commercial-scale cost-benefit analysis.

Future research should investigate the effects of this emulsifier blend on lipid digestibility, gut morphology, digestive physiology, and metabolic responses under different dietary fat sources and environmental conditions. Comprehensive economic assessments and commercial-scale validation studies are also warranted to determine the practical value of phase-specific emulsifier supplementation programs.

Overall, the results suggest that a phase-specific emulsifier supplementation strategy using a ternary blend of sorbitan esters, lysolecithin, and de-oiled lecithin is a promising nutritional approach for supporting growth performance and nutrient utilization in broilers fed reduced-energy diets. Strategic application of emulsifiers during periods of greatest physiological demand may enhance feeding efficiency and contribute to more sustainable and cost-effective broiler production.

## DATA AVAILABILITY

The data generated during the study are included in the manuscript.

## GENERATIVE AI DECLARATION

The authors declare that generative artificial intelligence (AI) tools were used solely to improve language, grammar, and readability during manuscript preparation. All scientific content, data analysis, interpretation of results, and conclusions were developed and verified by the authors. The authors take full responsibility for the accuracy, integrity, and originality of the work presented, and no AI tool was listed as an author.

## AUTHORS’ CONTRIBUTIONS

TP, PK, SP, DW, and SZ: Conceptualized and designed the study, supervised the project, conducted the experiments, managed data collection and analysis, and drafted and critically revised the manuscript. TP and SP: Performed sample collection and laboratory analyses. All authors have read and approved the final version of the manuscript.
